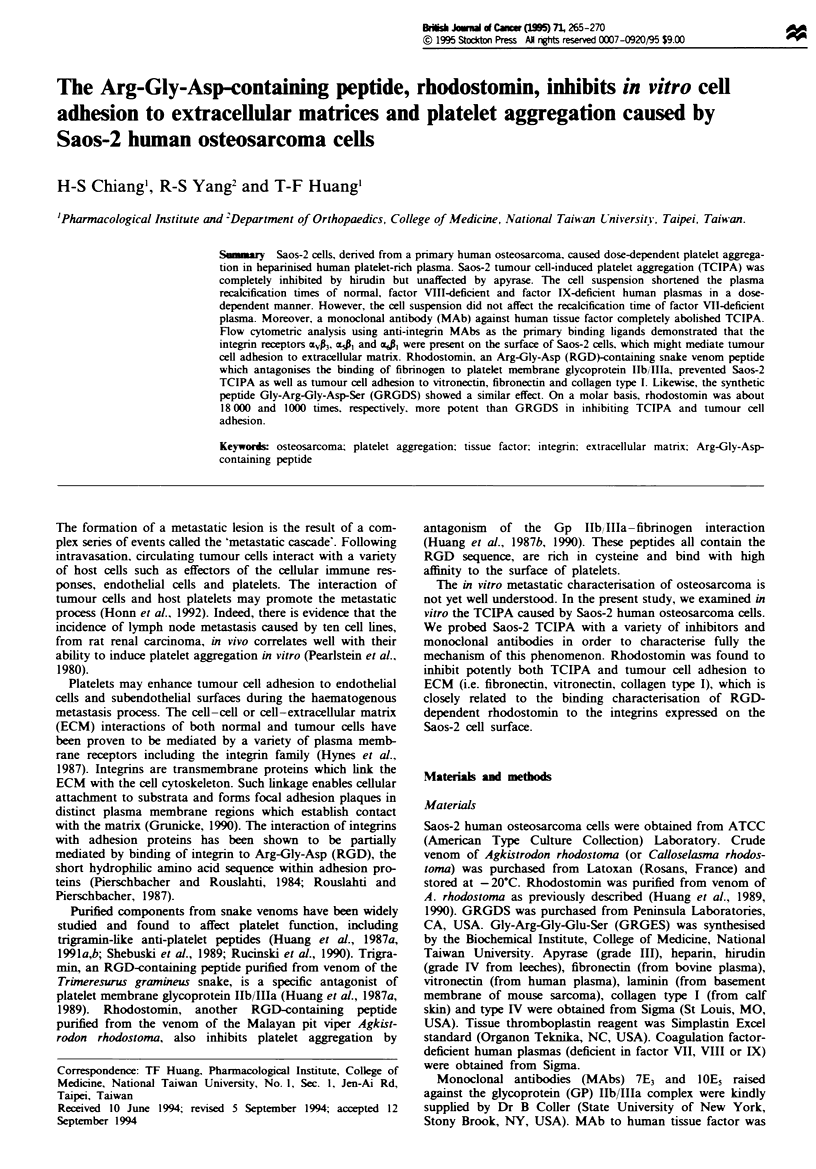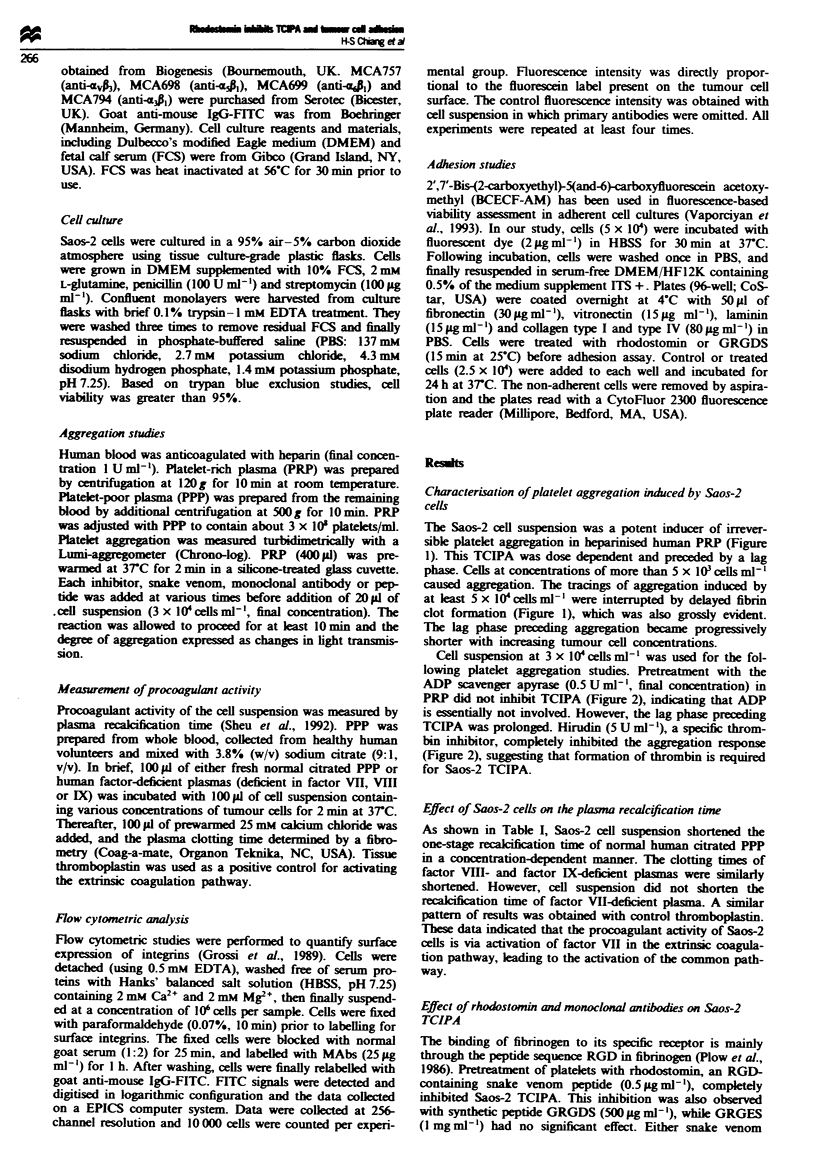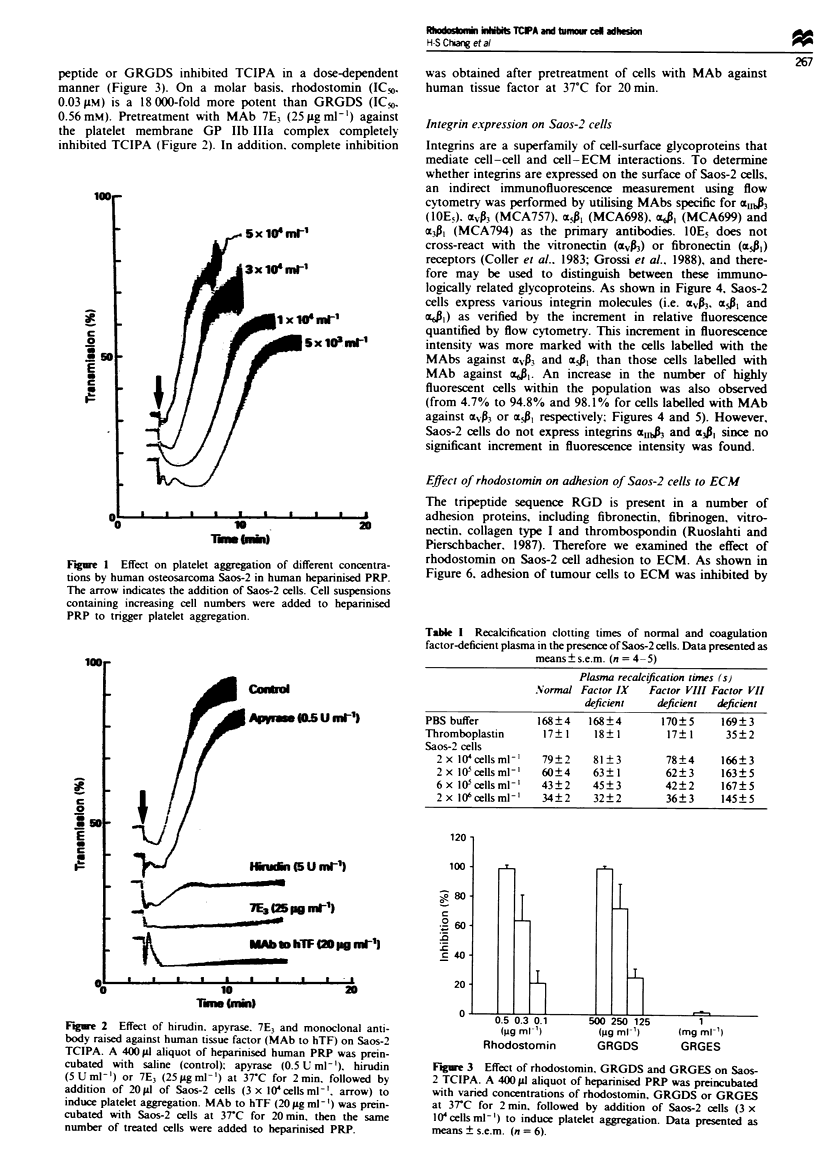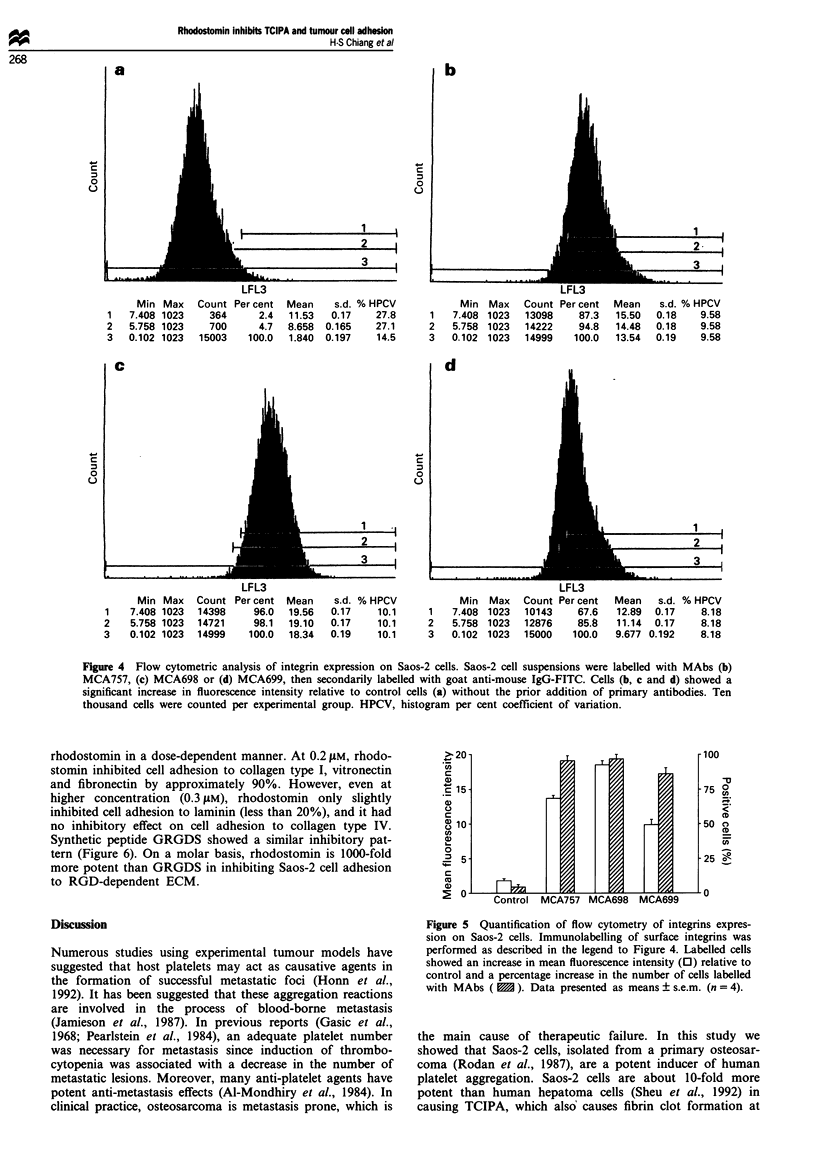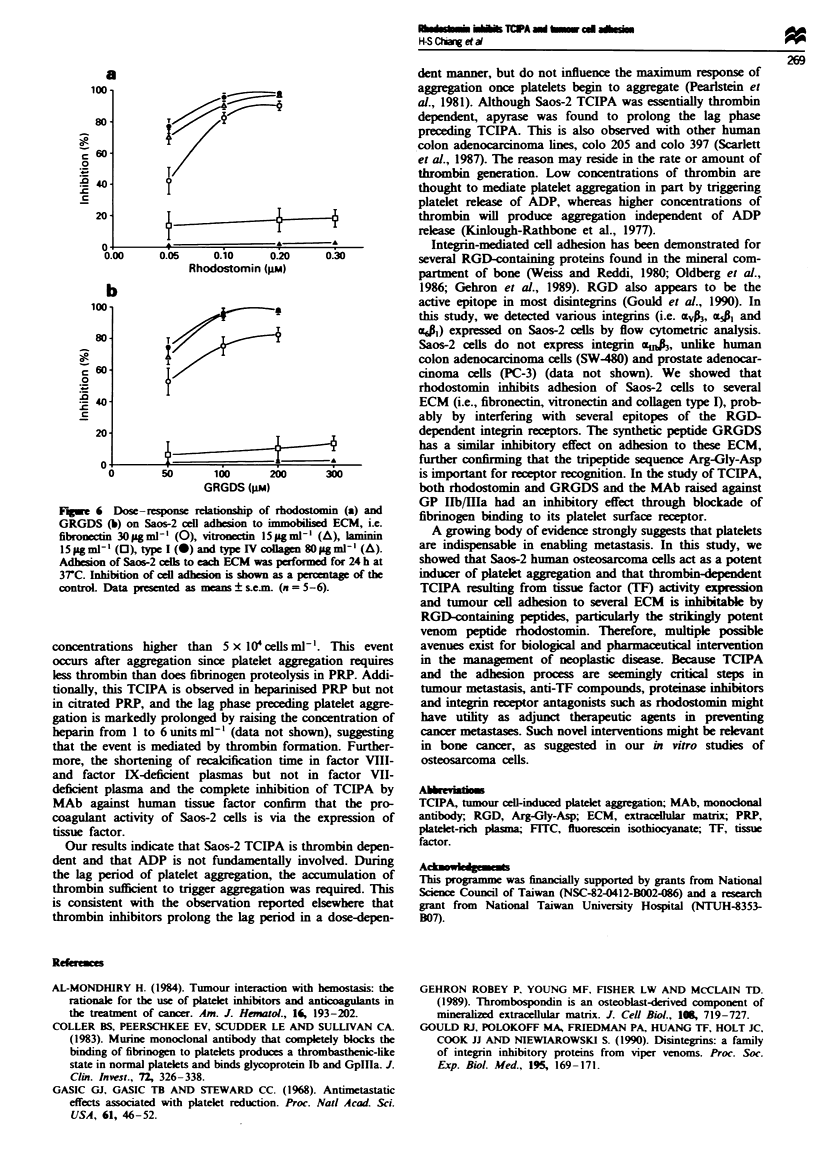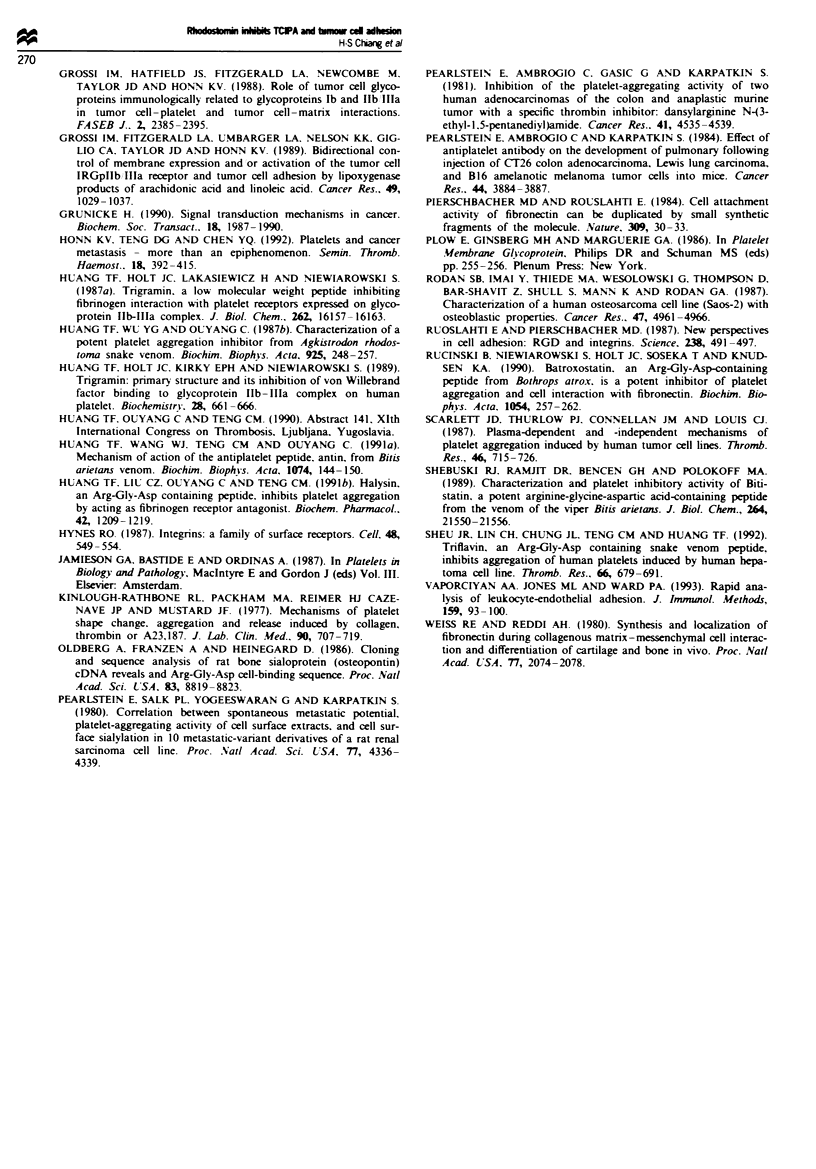# The Arg-Gly-Asp-containing peptide, rhodostomin, inhibits in vitro cell adhesion to extracellular matrices and platelet aggregation caused by saos-2 human osteosarcoma cells.

**DOI:** 10.1038/bjc.1995.54

**Published:** 1995-02

**Authors:** H. S. Chiang, R. S. Yang, T. F. Huang

**Affiliations:** Pharmacological Institute, College of Medicine, National Taiwan University, Taipei.

## Abstract

Saos-2 cells, derived from a primary human osteosarcoma, caused dose-dependent platelet aggregation in heparinised human platelet-rich plasma. Saos-2 tumour cell-induced platelet aggregation (TCIPA) was completely inhibited by hirudin but unaffected by apyrase. The cell suspension shortened the plasma recalcification times of normal, factor VIII-deficient and factor IX-deficient human plasmas in a dose-dependent manner. However, the cell suspension did not affect the recalcification time of factor VII-deficient plasma. Moreover, a monoclonal antibody (MAb) against human tissue factor completely abolished TCIPA. Flow cytometric analysis using anti-integrin MAbs as the primary binding ligands demonstrated that the integrin receptors alpha v beta 3, alpha 5 beta 1 and alpha 6 beta 1 were present of Saos-2 cells, which might mediate tumour cell adhesion to extracellular matrix. Rhodostomin, an Arg-Gly-Asp (RGD)-containing snake venom peptide which antagonises the binding of fibrinogen to platelet membrane glycoprotein IIb/IIIa, prevented Saos-2 TCIPA as well as tumour cell adhesion to vitronectin, fibronectin and collagen type I. Likewise, the synthetic peptide Gly-Arg-Gly-Asp-Ser (GRGDS) showed a similar effect. On a molar basis, rhodostomin was about 18,000 and 1000 times, respectively, more potent than GRGDS in inhibiting TCIPA and tumour cell adhesion.


British Journal of Cancer (1995) 71, 265-270

© 1995 Stockton Press All rights reserved 0007-0920/95 $9.00

### The Arg-Gly-Asp-containing peptide, rhodostomin, inhibits *in vitro* cell adhesion to extracellular matrices and platelet aggregation caused by Saos-2 human osteosarcoma cells

H-S Chiang[1], R-S Yang[2] and T-F Huang[1]

[1]*Pharmacological Institute and* [2]*Department of Orthopaedics, College of Medicine, National Taiwan University, Taipei, Taiwan.*

**Summary** Saos-2 cells, derived from a primary human osteosarcoma, caused dose-dependent platelet aggregation in heparinised human platelet-rich plasma. Saos-2 tumour cell-induced platelet aggregation (TCIPA) was completely inhibited by hirudin but unaffected by apyrase. The cell suspension shortened the plasma recalcification times of normal, factor VIII-deficient and factor IX-deficient human plasmas in a dose-dependent manner. However, the cell suspension did not affect the recalcification time of factor VII-deficient plasma. Moreover, a monoclonal antibody (MAb) against human tissue factor completely abolished TCIPA. Flow cytometric analysis using anti-integrin MAbs as the primary binding ligands demonstrated that the integrin receptors $\alpha_V\beta_3$, $\alpha_5\beta_1$ and $\alpha_6\beta_1$ were present on the surface of Saos-2 cells, which might mediate tumour cell adhesion to extracellular matrix. Rhodostomin, an Arg-Gly-Asp (RGD)-containing snake venom peptide which antagonises the binding of fibrinogen to platelet membrane glycoprotein IIb IIIa, prevented Saos-2 TCIPA as well as tumour cell adhesion to vitronectin, fibronectin and collagen type I. Likewise, the synthetic peptide Gly-Arg-Gly-Asp-Ser (GRGDS) showed a similar effect. On a molar basis, rhodostomin was about 18 000 and 1000 times, respectively, more potent than GRGDS in inhibiting TCIPA and tumour cell adhesion.

**Keywords:** osteosarcoma; platelet aggregation; tissue factor; integrin; extracellular matrix; Arg-Gly-Asp-containing peptide

The formation of a metastatic lesion is the result of a complex series of events called the 'metastatic cascade'. Following intravasation, circulating tumour cells interact with a variety of host cells such as effectors of the cellular immune responses, endothelial cells and platelets. The interaction of tumour cells and host platelets may promote the metastatic process (Honn *et al*., 1992). Indeed, there is evidence that the incidence of lymph node metastasis caused by ten cell lines, from rat renal carcinoma, *in vivo* correlates well with their ability to induce platelet aggregation *in vitro* (Pearlstein *et al*., 1980).

Platelets may enhance tumour cell adhesion to endothelial cells and subendothelial surfaces during the haematogenous metastasis process. The cell–cell or cell–extracellular matrix (ECM) interactions of both normal and tumour cells have been proven to be mediated by a variety of plasma membrane receptors including the integrin family (Hynes *et al*., 1987). Integrins are transmembrane proteins which link the ECM with the cell cytoskeleton. Such linkage enables cellular attachment to substrata and forms focal adhesion plaques in distinct plasma membrane regions which establish contact with the matrix (Grunicke, 1990). The interaction of integrins with adhesion proteins has been shown to be partially mediated by binding of integrin to Arg-Gly-Asp (RGD), the short hydrophilic amino acid sequence within adhesion proteins (Pierschbacher and Rouslahti, 1984; Rouslahti and Pierschbacher, 1987).

Purified components from snake venoms have been widely studied and found to affect platelet function, including trigramin-like anti-platelet peptides (Huang *et al*., 1987*a*, 1991*a,b*; Shebuski *et al*., 1989; Rucinski *et al*., 1990). Trigramin, an RGD-containing peptide purified from venom of the *Trimeresurus gramineus* snake, is a specific antagonist of platelet membrane glycoprotein IIb IIIa (Huang *et al*., 1987*a*, 1989). Rhodostomin, another RGD-containing peptide purified from the venom of the Malayan pit viper *Agkistrodon rhodostoma*, also inhibits platelet aggregation by antagonism of the Gp IIb IIIa–fibrinogen interaction (Huang *et al*., 1987*b*, 1990). These peptides all contain the RGD sequence, are rich in cysteine and bind with high affinity to the surface of platelets.

The *in vitro* metastatic characterisation of osteosarcoma is not yet well understood. In the present study, we examined *in vitro* the TCIPA caused by Saos-2 human osteosarcoma cells. We probed Saos-2 TCIPA with a variety of inhibitors and monoclonal antibodies in order to characterise fully the mechanism of this phenomenon. Rhodostomin was found to inhibit potently both TCIPA and tumour cell adhesion to ECM (i.e. fibronectin, vitronectin, collagen type I), which is closely related to the binding characterisation of RGD-dependent rhodostomin to the integrins expressed on the Saos-2 cell surface.

### Materials and methods

### *Materials*

Saos-2 human osteosarcoma cells were obtained from ATCC (American Type Culture Collection) Laboratory. Crude venom of *Agkistrodon rhodostoma* (or *Calloselasma rhodostoma*) was purchased from Latoxan (Rosans, France) and stored at −20°C. Rhodostomin was purified from venom of *A. rhodostoma* as previously described (Huang *et al*., 1989, 1990). GRGDS was purchased from Peninsula Laboratories, CA, USA. Gly-Arg-Gly-Glu-Ser (GRGES) was synthesised by the Biochemical Institute, College of Medicine, National Taiwan University. Apyrase (grade III), heparin, hirudin (grade IV from leeches), fibronectin (from bovine plasma), vitronectin (from human plasma), laminin (from basement membrane of mouse sarcoma), collagen type I (from calf skin) and type IV were obtained from Sigma (St Louis, MO, USA). Tissue thromboplastin reagent was Simplastin Excel standard (Organon Teknika, NC, USA). Coagulation factor-deficient human plasmas (deficient in factor VII, VIII or IX) were obtained from Sigma.

Monoclonal antibodies (MAbs) $7E_3$ and $10E_5$ raised against the glycoprotein (GP) IIb IIIa complex were kindly supplied by Dr B Coller (State University of New York, Stony Brook, NY, USA). MAb to human tissue factor was

Correspondence: TF Huang, Pharmacological Institute, College of Medicine, National Taiwan University, No. 1, Sec. 1, Jen-Ai Rd, Taipei, Taiwan
Received 10 June 1994; revised 5 September 1994; accepted 12 September 1994

obtained from Biogenesis (Bournemouth, UK. MCA757 (anti-$\alpha_V\beta_3$), MCA698 (anti-$\alpha_5\beta_1$), MCA699 (anti-$\alpha_6\beta_1$) and MCA794 (anti-$\alpha_3\beta_1$) were purchased from Serotec (Bicester, UK). Goat anti-mouse IgG-FITC was from Boehringer (Mannheim, Germany). Cell culture reagents and materials, including Dulbecco's modified Eagle medium (DMEM) and fetal calf serum (FCS) were from Gibco (Grand Island, NY, USA). FCS was heat inactivated at 56°C for 30 min prior to use.

*Cell culture*

Saos-2 cells were cultured in a 95% air–5% carbon dioxide atmosphere using tissue culture-grade plastic flasks. Cells were grown in DMEM supplemented with 10% FCS, 2 mM L-glutamine, penicillin (100 U $ml^{-1}$) and streptomycin (100 μg $ml^{-1}$). Confluent monolayers were harvested from culture flasks with brief 0.1% trypsin–1 mM EDTA treatment. They were washed three times to remove residual FCS and finally resuspended in phosphate-buffered saline (PBS: 137 mM sodium chloride, 2.7 mM potassium chloride, 4.3 mM disodium hydrogen phosphate, 1.4 mM potassium phosphate, pH 7.25). Based on trypan blue exclusion studies, cell viability was greater than 95%.

*Aggregation studies*

Human blood was anticoagulated with heparin (final concentration 1 U $ml^{-1}$). Platelet-rich plasma (PRP) was prepared by centrifugation at 120 *g* for 10 min at room temperature. Platelet-poor plasma (PPP) was prepared from the remaining blood by additional centrifugation at 500 *g* for 10 min. PRP was adjusted with PPP to contain about $3 \times 10^8$ platelets ml. Platelet aggregation was measured turbidimetrically with a Lumi-aggregometer (Chrono-log). PRP (400 μl) was prewarmed at 37°C for 2 min in a silicone-treated glass cuvette. Each inhibitor, snake venom, monoclonal antibody or peptide was added at various times before addition of 20 μl of cell suspension ($3 \times 10^4$ cells $ml^{-1}$, final concentration). The reaction was allowed to proceed for at least 10 min and the degree of aggregation expressed as changes in light transmission.

*Measurement of procoagulant activity*

Procoagulant activity of the cell suspension was measured by plasma recalcification time (Sheu *et al.*, 1992). PPP was prepared from whole blood, collected from healthy human volunteers and mixed with 3.8% (w v) sodium citrate (9:1, v v). In brief, 100 μl of either fresh normal citrated PPP or human factor-deficient plasmas (deficient in factor VII, VIII or IX) was incubated with 100 μl of cell suspension containing various concentrations of tumour cells for 2 min at 37°C. Thereafter, 100 μl of prewarmed 25 mM calcium chloride was added, and the plasma clotting time determined by a fibrometry (Coag-a-mate, Organon Teknika, NC, USA). Tissue thromboplastin was used as a positive control for activating the extrinsic coagulation pathway.

*Flow cytometric analysis*

Flow cytometric studies were performed to quantify surface expression of integrins (Grossi *et al.*, 1989). Cells were detached (using 0.5 mM EDTA), washed free of serum proteins with Hanks' balanced salt solution (HBSS, pH 7.25) containing 2 mM $Ca^{2+}$ and 2 mM $Mg^{2+}$, then finally suspended at a concentration of $10^6$ cells per sample. Cells were fixed with paraformaldehyde (0.07%, 10 min) prior to labelling for surface integrins. The fixed cells were blocked with normal goat serum (1:2) for 25 min, and labelled with MAbs (25 μg $ml^{-1}$) for 1 h. After washing, cells were finally relabelled with goat anti-mouse IgG-FITC. FITC signals were detected and digitised in logarithmic configuration and the data collected on a EPICS computer system. Data were collected at 256-channel resolution and 10 000 cells were counted per experimental group. Fluorescence intensity was directly proportional to the fluorescein label present on the tumour cell surface. The control fluorescence intensity was obtained with cell suspension in which primary antibodies were omitted. All experiments were repeated at least four times.

*Adhesion studies*

2',7'-Bis-(2-carboxyethyl)-5(and-6)-carboxyfluorescein acetoxymethyl (BCECF-AM) has been used in fluorescence-based viability assessment in adherent cell cultures (Vaporciyan *et al.*, 1993). In our study, cells ($5 \times 10^4$) were incubated with fluorescent dye (2 μg $ml^{-1}$) in HBSS for 30 min at 37°C. Following incubation, cells were washed once in PBS, and finally resuspended in serum-free DMEM HF12K containing 0.5% of the medium supplement ITS +. Plates (96-well: CoStar, USA) were coated overnight at 4°C with 50 μl of fibronectin (30 μg $ml^{-1}$), vitronectin (15 μg $ml^{-1}$), laminin (15 μg $ml^{-1}$) and collagen type I and type IV (80 μg $ml^{-1}$) in PBS. Cells were treated with rhodostomin or GRGDS (15 min at 25°C) before adhesion assay. Control or treated cells ($2.5 \times 10^4$) were added to each well and incubated for 24 h at 37°C. The non-adherent cells were removed by aspiration and the plates read with a CytoFluor 2300 fluorescence plate reader (Millipore, Bedford, MA, USA).

### Results

*Characterisation of platelet aggregation induced by Saos-2 cells*

The Saos-2 cell suspension was a potent inducer of irreversible platelet aggregation in heparinised human PRP (Figure 1). This TCIPA was dose dependent and preceded by a lag phase. Cells at concentrations of more than $5 \times 10^3$ cells $ml^{-1}$ caused aggregation. The tracings of aggregation induced by at least $5 \times 10^4$ cells $ml^{-1}$ were interrupted by delayed fibrin clot formation (Figure 1), which was also grossly evident. The lag phase preceding aggregation became progressively shorter with increasing tumour cell concentrations.

Cell suspension at $3 \times 10^4$ cells $ml^{-1}$ was used for the following platelet aggregation studies. Pretreatment with the ADP scavenger apyrase (0.5 U $ml^{-1}$, final concentration) in PRP did not inhibit TCIPA (Figure 2), indicating that ADP is essentially not involved. However, the lag phase preceding TCIPA was prolonged. Hirudin (5 U $ml^{-1}$), a specific thrombin inhibitor, completely inhibited the aggregation response (Figure 2), suggesting that formation of thrombin is required for Saos-2 TCIPA.

*Effect of Saos-2 cells on the plasma recalcification time*

As shown in Table I, Saos-2 cell suspension shortened the one-stage recalcification time of normal human citrated PPP in a concentration-dependent manner. The clotting times of factor VIII- and factor IX-deficient plasmas were similarly shortened. However, cell suspension did not shorten the recalcification time of factor VII-deficient plasma. A similar pattern of results was obtained with control thromboplastin. These data indicated that the procoagulant activity of Saos-2 cells is via activation of factor VII in the extrinsic coagulation pathway, leading to the activation of the common pathway.

*Effect of rhodostomin and monoclonal antibodies on Saos-2 TCIPA*

The binding of fibrinogen to its specific receptor is mainly through the peptide sequence RGD in fibrinogen (Plow *et al.*, 1986). Pretreatment of platelets with rhodostomin, an RGD-containing snake venom peptide (0.5 μg $ml^{-1}$), completely inhibited Saos-2 TCIPA. This inhibition was also observed with synthetic peptide GRGDS (500 μg $ml^{-1}$), while GRGES (1 mg $ml^{-1}$) had no significant effect. Either snake venom peptide or GRGDS inhibited TCIPA in a dose-dependent manner (Figure 3). On a molar basis, rhodostomin ($IC_{50}$, 0.03 μM) is a 18 000-fold more potent than GRGDS ($IC_{50}$, 0.56 mM). Pretreatment with MAb $7E_3$ (25 μg $ml^{-1}$) against the platelet membrane GP IIb IIIa complex completely inhibited TCIPA (Figure 2). In addition, complete inhibition was obtained after pretreatment of cells with MAb against human tissue factor at 37°C for 20 min.

**Figure 1** Effect on platelet aggregation of different concentrations by human osteosarcoma Saos-2 in human heparinised PRP. The arrow indicates the addition of Saos-2 cells. Cell suspensions containing increasing cell numbers were added to heparinised PRP to trigger platelet aggregation.

**Figure 2** Effect of hirudin, apyrase, $7E_3$ and monoclonal antibody raised against human tissue factor (MAb to hTF) on Saos-2 TCIPA. A 400 μl aliquot of heparinised human PRP was preincubated with saline (control); apyrase (0.5 U $ml^{-1}$), hirudin (5 U $ml^{-1}$) or $7E_3$ (25 μg $ml^{-1}$) at 37°C for 2 min, followed by addition of 20 μl of Saos-2 cells ($3 \times 10^4$ cells $ml^{-1}$, arrow) to induce platelet aggregation. MAb to hTF (20 μg $ml^{-1}$) was preincubated with Saos-2 cells at 37°C for 20 min, then the same number of treated cells were added to heparinised PRP.

*Integrin expression on Saos-2 cells*

Integrins are a superfamily of cell-surface glycoproteins that mediate cell–cell and cell–ECM interactions. To determine whether integrins are expressed on the surface of Saos-2 cells, an indirect immunofluorescence measurement using flow cytometry was performed by utilising MAbs specific for $\alpha_{IIb}\beta_3$ ($10E_5$), $\alpha_V\beta_3$ (MCA757), $\alpha_5\beta_1$ (MCA698), $\alpha_6\beta_1$ (MCA699) and $\alpha_3\beta_1$ (MCA794) as the primary antibodies. $10E_5$ does not cross-react with the vitronectin ($\alpha_V\beta_3$) or fibronectin ($\alpha_5\beta_1$) receptors (Coller *et al.*, 1983; Grossi *et al.*, 1988), and therefore may be used to distinguish between these immunologically related glycoproteins. As shown in Figure 4, Saos-2 cells express various integrin molecules (i.e. $\alpha_V\beta_3$, $\alpha_5\beta_1$ and $\alpha_6\beta_1$) as verified by the increment in relative fluorescence quantified by flow cytometry. This increment in fluorescence intensity was more marked with the cells labelled with the MAbs against $\alpha_V\beta_3$ and $\alpha_5\beta_1$ than those cells labelled with MAb against $\alpha_6\beta_1$. An increase in the number of highly fluorescent cells within the population was also observed (from 4.7% to 94.8% and 98.1% for cells labelled with MAb against $\alpha_V\beta_3$ or $\alpha_5\beta_1$ respectively; Figures 4 and 5). However, Saos-2 cells do not express integrins $\alpha_{IIb}\beta_3$ and $\alpha_3\beta_1$ since no significant increment in fluorescence intensity was found.

*Effect of rhodostomin on adhesion of Saos-2 cells to ECM*

The tripeptide sequence RGD is present in a number of adhesion proteins, including fibronectin, fibrinogen, vitronectin, collagen type I and thrombospondin (Ruoslahti and Pierschbacher, 1987). Therefore we examined the effect of rhodostomin on Saos-2 cell adhesion to ECM. As shown in Figure 6, adhesion of tumour cells to ECM was inhibited by

**Table I** Recalcification clotting times of normal and coagulation factor-deficient plasma in the presence of Saos-2 cells. Data presented as means ± s.e.m. ($n = 4–5$)

| | *Plasma recalcification times (s)* | | | |
|---|---|---|---|---|
| | *Normal* | *Factor IX deficient* | *Factor VIII deficient* | *Factor VII deficient* |
| PBS buffer | 168±4 | 168±4 | 170±5 | 169±3 |
| Thromboplastin | 17±1 | 18±1 | 17±1 | 35±2 |
| Saos-2 cells | | | | |
| $2 \times 10^4$ cells $ml^{-1}$ | 79±2 | 81±3 | 78±4 | 166±3 |
| $2 \times 10^5$ cells $ml^{-1}$ | 60±4 | 63±1 | 62±3 | 163±5 |
| $6 \times 10^5$ cells $ml^{-1}$ | 43±2 | 45±3 | 42±2 | 167±5 |
| $2 \times 10^6$ cells $ml^{-1}$ | 34±2 | 32±2 | 36±3 | 145±5 |

**Figure 3** Effect of rhodostomin, GRGDS and GRGES on Saos-2 TCIPA. A 400 μl aliquot of heparinised PRP was preincubated with varied concentrations of rhodostomin, GRGDS or GRGES at 37°C for 2 min, followed by addition of Saos-2 cells ($3 \times 10^4$ cells $ml^{-1}$) to induce platelet aggregation. Data presented as means ± s.e.m. ($n = 6$).

a

| | Min | Max | Count | Per cent | Mean | s.d. | % HPCV |
|---|---|---|---|---|---|---|---|
| 1 | 7.408 | 1023 | 364 | 2.4 | 11.53 | 0.17 | 27.8 |
| 2 | 5.758 | 1023 | 700 | 4.7 | 8.658 | 0.165 | 27.1 |
| 3 | 0.102 | 1023 | 15003 | 100.0 | 1.840 | 0.197 | 14.5 |

b

| | Min | Max | Count | Per cent | Mean | s.d. | % HPCV |
|---|---|---|---|---|---|---|---|
| 1 | 7.408 | 1023 | 13098 | 87.3 | 15.50 | 0.18 | 9.58 |
| 2 | 5.758 | 1023 | 14222 | 94.8 | 14.48 | 0.18 | 9.58 |
| 3 | 0.102 | 1023 | 14999 | 100.0 | 13.54 | 0.19 | 9.58 |

c

| | Min | Max | Count | Per cent | Mean | s.d. | % HPCV |
|---|---|---|---|---|---|---|---|
| 1 | 7.408 | 1023 | 14398 | 96.0 | 19.56 | 0.17 | 10.1 |
| 2 | 5.758 | 1023 | 14721 | 98.1 | 19.10 | 0.17 | 10.1 |
| 3 | 0.102 | 1023 | 14999 | 100.0 | 18.34 | 0.19 | 10.1 |

d

| | Min | Max | Count | Per cent | Mean | s.d. | % HPCV |
|---|---|---|---|---|---|---|---|
| 1 | 7.408 | 1023 | 10143 | 67.6 | 12.89 | 0.17 | 8.18 |
| 2 | 5.758 | 1023 | 12876 | 85.8 | 11.14 | 0.17 | 8.18 |
| 3 | 0.102 | 1023 | 15000 | 100.0 | 9.677 | 0.192 | 8.18 |

rhodostomin in a dose-dependent manner. At 0.2 μM, rhodostomin inhibited cell adhesion to collagen type I, vitronectin and fibronectin by approximately 90%. However, even at higher concentration (0.3 μM), rhodostomin only slightly inhibited cell adhesion to laminin (less than 20%), and it had no inhibitory effect on cell adhesion to collagen type IV. Synthetic peptide GRGDS showed a similar inhibitory pattern (Figure 6). On a molar basis, rhodostomin is 1000-fold more potent than GRGDS in inhibiting Saos-2 cell adhesion to RGD-dependent ECM.

**Figure 5** Quantification of flow cytometry of integrins expression on Saos-2 cells. Immunolabelling of surface integrins was performed as described in the legend to Figure 4. Labelled cells showed an increase in mean fluorescence intensity (□) relative to control and a percentage increase in the number of cells labelled with MAbs (▨). Data presented as means ± s.e.m. ($n = 4$).

### Discussion

Numerous studies using experimental tumour models have suggested that host platelets may act as causative agents in the formation of successful metastatic foci (Honn *et al.*, 1992). It has been suggested that these aggregation reactions are involved in the process of blood-borne metastasis (Jamieson *et al.*, 1987). In previous reports (Gasic *et al.*, 1968; Pearlstein *et al.*, 1984), an adequate platelet number was necessary for metastasis since induction of thrombocytopenia was associated with a decrease in the number of metastatic lesions. Moreover, many anti-platelet agents have potent anti-metastasis effects (Al-Mondhiry *et al.*, 1984). In clinical practice, osteosarcoma is metastasis prone, which is the main cause of therapeutic failure. In this study we showed that Saos-2 cells, isolated from a primary osteosarcoma (Rodan *et al.*, 1987), are a potent inducer of human platelet aggregation. Saos-2 cells are about 10-fold more potent than human hepatoma cells (Sheu *et al.*, 1992) in causing TCIPA, which also causes fibrin clot formation at

**Figure 6** Dose-response relationship of rhodostomin (**a**) and GRGDS (**b**) on Saos-2 cell adhesion to immobilised ECM, i.e. fibronectin 30 μg ml$^{-1}$ (○), vitronectin 15 μg ml$^{-1}$ (Δ), laminin 15 μg ml$^{-1}$ (□), type I (●) and type IV collagen 80 μg ml$^{-1}$ (Δ). Adhesion of Saos-2 cells to each ECM was performed for 24 h at 37°C. Inhibition of cell adhesion is shown as a percentage of the control. Data presented as means ± s.e.m. ($n$ = 5-6).

concentrations higher than $5 \times 10^4$ cells ml$^{-1}$. This event occurs after aggregation since platelet aggregation requires less thrombin than does fibrinogen proteolysis in PRP. Additionally, this TCIPA is observed in heparinised PRP but not in citrated PRP, and the lag phase preceding platelet aggregation is markedly prolonged by raising the concentration of heparin from 1 to 6 units ml$^{-1}$ (data not shown), suggesting that the event is mediated by thrombin formation. Furthermore, the shortening of recalcification time in factor VIII- and factor IX-deficient plasmas but not in factor VII-deficient plasma and the complete inhibition of TCIPA by MAb against human tissue factor confirm that the procoagulant activity of Saos-2 cells is via the expression of tissue factor.

Our results indicate that Saos-2 TCIPA is thrombin dependent and that ADP is not fundamentally involved. During the lag period of platelet aggregation, the accumulation of thrombin sufficient to trigger aggregation was required. This is consistent with the observation reported elsewhere that thrombin inhibitors prolong the lag period in a dose-dependent manner, but do not influence the maximum response of aggregation once platelets begin to aggregate (Pearlstein *et al.*, 1981). Although Saos-2 TCIPA was essentially thrombin dependent, apyrase was found to prolong the lag phase preceding TCIPA. This is also observed with other human colon adenocarcinoma lines, colo 205 and colo 397 (Scarlett *et al.*, 1987). The reason may reside in the rate or amount of thrombin generation. Low concentrations of thrombin are thought to mediate platelet aggregation in part by triggering platelet release of ADP, whereas higher concentrations of thrombin will produce aggregation independent of ADP release (Kinlough-Rathbone et al., 1977).

Integrin-mediated cell adhesion has been demonstrated for several RGD-containing proteins found in the mineral compartment of bone (Weiss and Reddi, 1980; Oldberg *et al.*, 1986; Gehron *et al.*, 1989). RGD also appears to be the active epitope in most disintegrins (Gould *et al.*, 1990). In this study, we detected various integrins (i.e. $\alpha_v\beta_3$, $\alpha_5\beta_1$ and $\alpha_6\beta_1$) expressed on Saos-2 cells by flow cytometric analysis. Saos-2 cells do not express integrin $\alpha_{IIb}\beta_3$, unlike human colon adenocarcinoma cells (SW-480) and prostate adenocarcinoma cells (PC-3) (data not shown). We showed that rhodostomin inhibits adhesion of Saos-2 cells to several ECM (i.e., fibronectin, vitronectin and collagen type I), probably by interfering with several epitopes of the RGD-dependent integrin receptors. The synthetic peptide GRGDS has a similar inhibitory effect on adhesion to these ECM, further confirming that the tripeptide sequence Arg-Gly-Asp is important for receptor recognition. In the study of TCIPA, both rhodostomin and GRGDS and the MAb raised against GP IIb IIIa had an inhibitory effect through blockade of fibrinogen binding to its platelet surface receptor.

A growing body of evidence strongly suggests that platelets are indispensable in enabling metastasis. In this study, we showed that Saos-2 human osteosarcoma cells act as a potent inducer of platelet aggregation and that thrombin-dependent TCIPA resulting from tissue factor (TF) activity expression and tumour cell adhesion to several ECM is inhibitable by RGD-containing peptides, particularly the strikingly potent venom peptide rhodostomin. Therefore, multiple possible avenues exist for biological and pharmaceutical intervention in the management of neoplastic disease. Because TCIPA and the adhesion process are seemingly critical steps in tumour metastasis, anti-TF compounds, proteinase inhibitors and integrin receptor antagonists such as rhodostomin might have utility as adjunct therapeutic agents in preventing cancer metastases. Such novel interventions might be relevant in bone cancer, as suggested in our *in vitro* studies of osteosarcoma cells.

**Abbreviations**

TCIPA, tumour cell-induced platelet aggregation; MAb, monoclonal antibody; RGD, Arg-Gly-Asp; ECM, extracellular matrix; PRP, platelet-rich plasma; FITC, fluorescein isothiocyanate; TF, tissue factor.

**Acknowledgements**

This programme was financially supported by grants from National Science Council of Taiwan (NSC-82-0412-B002-086) and a research grant from National Taiwan University Hospital (NTUH-8353-B07).

**References**

AL-MONDHIRY H. (1984). Tumour interaction with hemostasis: the rationale for the use of platelet inhibitors and anticoagulants in the treatment of cancer. *Am. J. Hematol.*, **16**, 193-202.

COLLER BS, PEERSCHKEE EV, SCUDDER LE AND SULLIVAN CA. (1983). Murine monoclonal antibody that completely blocks the binding of fibrinogen to platelets produces a thrombasthenic-like state in normal platelets and binds glycoprotein Ib and GpIIIa. *J. Clin. Invest.*, **72**, 326-338.

GASIC GJ, GASIC TB AND STEWARD CC. (1968). Antimetastatic effects associated with platelet reduction. *Proc. Natl Acad. Sci. USA*, **61**, 46-52.

GEHRON ROBEY P, YOUNG MF, FISHER LW AND McCLAIN TD. (1989). Thrombospondin is an osteoblast-derived component of mineralized extracellular matrix. *J. Cell Biol.*, **108**, 719-727.

GOULD RJ, POLOKOFF MA, FRIEDMAN PA, HUANG TF, HOLT JC, COOK JJ AND NIEWIAROWSKI S. (1990). Disintegrins: a family of integrin inhibitory proteins from viper venoms. *Proc. Soc. Exp. Biol. Med.*, **195**, 169-171.

GROSSI IM, HATFIELD JS, FITZGERALD LA, NEWCOMBE M, TAYLOR JD AND HONN KV. (1988). Role of tumor cell glycoproteins immunologically related to glycoproteins Ib and IIb/IIIa in tumor cell-platelet and tumor cell-matrix interactions. *FASEB J.*, **2**, 2385-2395.

GROSSI IM, FITZGERALD LA, UMBARGER LA, NELSON KK, GIGLIO CA, TAYLOR JD AND HONN KV. (1989). Bidirectional control of membrane expression and/or activation of the tumor cell IRGpIIb/IIIa receptor and tumor cell adhesion by lipoxygenase products of arachidonic acid and linoleic acid. *Cancer Res.*, **49**, 1029-1037.

GRUNICKE H. (1990). Signal transduction mechanisms in cancer. *Biochem. Soc. Transact.*, **18**, 1987-1990.

HONN KV, TENG DG AND CHEN YQ. (1992). Platelets and cancer metastasis - more than an epiphenomenon. *Semin. Thromb. Haemost.*, **18**, 392-415.

HUANG TF, HOLT JC, LAKASIEWICZ H AND NIEWIAROWSKI S. (1987*a*). Trigramin, a low molecular weight peptide inhibiting fibrinogen interaction with platelet receptors expressed on glycoprotein IIb-IIIa complex. *J. Biol. Chem.*, **262**, 16157-16163.

HUANG TF, WU YG AND OUYANG C. (1987*b*). Characterization of a potent platelet aggregation inhibitor from *Agkistrodon rhodostoma* snake venom. *Biochim. Biophys. Acta*, **925**, 248-257.

HUANG TF, HOLT JC, KIRKY EPH AND NIEWIAROWSKI S. (1989). Trigramin: primary structure and its inhibition of von Willebrand factor binding to glycoprotein IIb-IIIa complex on human platelet. *Biochemistry*, **28**, 661-666.

HUANG TF, OUYANG C AND TENG CM. (1990). Abstract 141, XIth International Congress on Thrombosis, Ljubljana, Yugoslavia.

HUANG TF, WANG WJ, TENG CM AND OUYANG C. (1991*a*). Mechanism of action of the antiplatelet peptide, antin, from *Bitis arietans* venom. *Biochim. Biophys. Acta*, **1074**, 144-150.

HUANG TF, LIU CZ, OUYANG C AND TENG CM. (1991*b*). Halysin, an Arg-Gly-Asp containing peptide, inhibits platelet aggregation by acting as fibrinogen receptor antagonist. *Biochem. Pharmacol.*, **42**, 1209-1219.

HYNES RO. (1987). Integrins: a family of surface receptors. *Cell*, **48**, 549-554.

JAMIESON GA, BASTIDE E AND ORDINAS A. (1987). In *Platelets in Biology and Pathology*, MacIntyre E and Gordon J (eds) Vol. III. Elsevier: Amsterdam.

KINLOUGH-RATHBONE RL, PACKHAM MA, REIMER HJ CAZENAVE JP AND MUSTARD JF. (1977). Mechanisms of platelet shape change, aggregation and release induced by collagen, thrombin or A23,187. *J. Lab. Clin. Med.*, **90**, 707-719.

OLDBERG A, FRANZEN A AND HEINEGARD D. (1986). Cloning and sequence analysis of rat bone sialoprotein (osteopontin) cDNA reveals and Arg-Gly-Asp cell-binding sequence. *Proc. Natl Acad. Sci. USA*, **83**, 8819-8823.

PEARLSTEIN E, SALK PL, YOGEESWARAN G AND KARPATKIN S. (1980). Correlation between spontaneous metastatic potential, platelet-aggregating activity of cell surface extracts, and cell surface sialylation in 10 metastatic-variant derivatives of a rat renal sarcinoma cell line. *Proc. Natl Acad. Sci. USA*, **77**, 4336-4339.

PEARLSTEIN E, AMBROGIO C, GASIC G AND KARPATKIN S. (1981). Inhibition of the platelet-aggregating activity of two human adenocarcinomas of the colon and anaplastic murine tumor with a specific thrombin inhibitor: dansylarginine N-(3-ethyl-1,5-pentanediyl)amide. *Cancer Res.*, **41**, 4535-4539.

PEARLSTEIN E, AMBROGIO C AND KARPATKIN S. (1984). Effect of antiplatelet antibody on the development of pulmonary following injection of CT26 colon adenocarcinoma, Lewis lung carcinoma, and B16 amelanotic melanoma tumor cells into mice. *Cancer Res.*, **44**, 3884-3887.

PIERSCHBACHER MD AND ROUSLAHTI E. (1984). Cell attachment activity of fibronectin can be duplicated by small synthetic fragments of the molecule. *Nature*, **309**, 30-33.

PLOW E, GINSBERG MH AND MARGUERIE GA. (1986). In *Platelet Membrane Glycoprotein*, Philips DR and Schuman MS (eds) pp. 255-256. Plenum Press: New York.

RODAN SB, IMAI Y, THIEDE MA, WESOLOWSKI G, THOMPSON D, BAR-SHAVIT Z, SHULL S, MANN K AND RODAN GA. (1987). Characterization of a human osteosarcoma cell line (Saos-2) with osteoblastic properties. *Cancer Res.*, **47**, 4961-4966.

RUOSLAHTI E AND PIERSCHBACHER MD. (1987). New perspectives in cell adhesion: RGD and integrins. *Science*, **238**, 491-497.

RUCINSKI B, NIEWIAROWSKI S, HOLT JC, SOSEKA T AND KNUDSEN KA. (1990). Batroxostatin, an Arg-Gly-Asp-containing peptide from *Bothrops atrox*, is a potent inhibitor of platelet aggregation and cell interaction with fibronectin. *Biochim. Biophys. Acta*, **1054**, 257-262.

SCARLETT JD, THURLOW PJ, CONNELLAN JM AND LOUIS CJ. (1987). Plasma-dependent and -independent mechanisms of platelet aggregation induced by human tumor cell lines. *Thromb. Res.*, **46**, 715-726.

SHEBUSKI RJ, RAMJIT DR, BENCEN GH AND POLOKOFF MA. (1989). Characterization and platelet inhibitory activity of Bitistatin, a potent arginine-glycine-aspartic acid-containing peptide from the venom of the viper *Bitis arietans*. *J. Biol. Chem.*, **264**, 21550-21556.

SHEU JR, LIN CH, CHUNG JL, TENG CM AND HUANG TF. (1992). Triflavin, an Arg-Gly-Asp containing snake venom peptide, inhibits aggregation of human platelets induced by human hepatoma cell line. *Thromb. Res.*, **66**, 679-691.

VAPORCIYAN AA, JONES ML AND WARD PA. (1993). Rapid analysis of leukocyte-endothelial adhesion. *J. Immunol. Methods*, **159**, 93-100.

WEISS RE AND REDDI AH. (1980). Synthesis and localization of fibronectin during collagenous matrix-messenchymal cell interaction and differentiation of cartilage and bone in vivo. *Proc. Natl Acad. USA*, **77**, 2074-2078.